# Targeting translocator protein (18 kDa) (TSPO) dampens pro-inflammatory microglia reactivity in the retina and protects from degeneration

**DOI:** 10.1186/s12974-015-0422-5

**Published:** 2015-11-02

**Authors:** Rebecca Scholz, Albert Caramoy, Mohajeet B. Bhuckory, Khalid Rashid, Mei Chen, Heping Xu, Christian Grimm, Thomas Langmann

**Affiliations:** Department of Ophthalmology, Laboratory for Experimental Immunology of the Eye, University of Cologne, 50931 Cologne, Germany; Centre for Experimental Medicine, School of Medicine, Dentistry and Biomedical Sciences, Queen’s University Belfast, Belfast, BT12 6BA UK; Department of Ophthalmology, Lab for Retinal Cell Biology, University of Zürich, 8057 Zürich, Switzerland

**Keywords:** Translocator protein (18 kDa) (TSPO), Microglia, Photoreceptors, Retinal degeneration, Light damage, Age-related macular degeneration

## Abstract

**Background:**

Reactive microglia are commonly seen in retinal degenerative diseases, and neurotoxic microglia responses can contribute to photoreceptor cell death. We and others have previously shown that translocator protein (18 kDa) (TSPO) is highly induced in retinal degenerations and that the selective TSPO ligand XBD173 (AC-5216, emapunil) exerts strong anti-inflammatory effects on microglia in vitro and ex vivo. Here, we investigated whether targeting TSPO with XBD173 has immuno-modulatory and neuroprotective functions in two mouse models of acute retinal degeneration using bright white light exposure.

**Methods:**

BALB/cJ and *Cx3cr1*^GFP/+^ mice received intraperitoneal injections of 10 mg/kg XBD173 or vehicle for five consecutive days, starting 1 day prior to exposure to either 15,000 lux white light for 1 h or 50,000 lux focal light for 10 min, respectively. The effects of XBD173 treatment on microglia and Müller cell reactivity were analyzed by immuno-stainings of retinal sections and flat mounts, fluorescence-activated cell sorting (FACS) analysis, and mRNA expression of microglia markers using quantitative real-time PCR (qRT-PCR). Optical coherence tomography (OCT), terminal deoxynucleotidyl transferase dUTP nick end labeling (TUNEL) stainings, and morphometric analyses were used to quantify the extent of retinal degeneration and photoreceptor apoptosis.

**Results:**

Four days after the mice were challenged with bright white light, a large number of amoeboid-shaped alerted microglia appeared in the degenerating outer retina, which was nearly completely prevented by treatment with XBD173. This treatment also down-regulated the expression of TSPO protein in microglia but did not change the TSPO levels in the retinal pigment epithelium (RPE). RT-PCR analysis showed that the microglia/macrophage markers Cd68 and activated microglia/macrophage whey acidic protein (Amwap) as well as the pro-inflammatory genes *Ccl2* and *Il6* were reduced after XBD173 treatment. Light-induced degeneration of the outer retina was nearly fully blocked by XBD173 treatment. We further confirmed these findings in an independent mouse model of focal light damage. Retinas of animals receiving XBD173 therapy displayed significantly more ramified non-reactive microglia and more viable arrestin-positive cone photoreceptors than vehicle controls.

**Conclusions:**

Targeting TSPO with XBD173 effectively counter-regulates microgliosis and ameliorates light-induced retinal damage, highlighting a new pharmacological concept for the treatment of retinal degenerations.

## Background

Microglial cells are the resident tissue macrophages of the central nervous system (CNS), including the retina. In the healthy retina, they are located in the plexiform layers from where they permanently scan the retinal environment with their motile protrusions [1]. Several receptors that are specific for the binding of chemokines, cytokines, complement factors, antibodies, or damage-associated molecular patterns enable these cells to recognize and immediately respond to pathological changes of their environment [2–4]. Beside their homeostatic function in the healthy retina, microglia reactivity and age-related changes of microglia physiology contribute to degenerative pathologies of the retina and the entire CNS [3, 5–14]. Together with monocytes and macrophages, microglial cells are major players in chronic immune processes including parainflammation [15]. Reactive microglia are detectable in the damaged photoreceptor layers of patients with degenerative retinal diseases such as retinitis pigmentosa and age-related macular degeneration (AMD) [6]. Microglia not only phagocytose dead cells but also take up living rods in a mouse model for retinitis pigmentosa [10]. Therefore, microglial activation cannot be just regarded as a bystander effect but rather actively contributes to photoreceptor cell death during retinal degeneration.

In attempts to better characterize retinal microglia reactivity and find novel markers, we and others have previously identified very high expression of translocator protein (18 kDa) in reactive retinal microglia [16, 17]. Translocator protein (TSPO), previously known as the peripheral benzodiazepine receptor, likely mediates the transport of cholesterol into the inner mitochondrial membrane, where it serves as a precursor for steroids and neurosteroids [18]. The protein is constitutively expressed in steroidogenic tissues and up-regulated in activated glial cells [19–21]. Glial up-regulation of TSPO is a major hallmark of neurodegenerative diseases [22], and various TSPO ligands have been developed as molecular markers to detect gliosis by means of PET imaging [23].

Specific TSPO ligands are also under investigation as treatment options for neurological disorders including Alzheimer’s disease [24], multiple sclerosis [25], neuropathic pain [26], peripheral nerve injury [22], and anxiety disorders [27]. Classical synthetic TSPO ligands such as the benzodiazepine derivative 4′-chlorodiazepam (Ro5-4864) and the isoquinoline carboxamide PK11195 directly enhance GABAergic neurotransmission [28]. In contrast, structurally different synthetic TSPO ligands such as etifoxine (Stresam) and XBD173 (AC-5216, emapunil) also stimulate the synthesis of neurosteroids and exert potent anti-inflammatory and neuroprotective effects [29]. XBD173 is a very selective and high-affinity phenylpurine ligand for TSPO whereas the benzoxazine etifoxine additionally binds GABA_A_ receptors [30]. Since XBD173 has a high and specific affinity for TSPO with a more beneficial side-effect profile than benzodiazepine derivatives, a precise and potentially long-term application to limit neuroinflammation seems feasible.

In a previous report, we have comprehensively characterized the anti-inflammatory effects of the TSPO ligand XBD173 using murine and human microglial cells as well as cultured mouse retinal explants [16]. XBD173 strongly suppressed pro-inflammatory gene expression in LPS-challenged microglia and diminished their neurotoxic potential on photoreceptor cell cultures, indicating that targeting TSPO with XBD173 is a promising approach to control microglial reactivity [16]. In this study, we addressed the questions whether XBD173 influences microglial reactivity in vitro and protects from acute white light-induced retinal degeneration in two different mouse models. We selected white light exposure as it is an environmental risk factor and mimics several features of human retinal degenerative diseases in rodents [31–33]. This model is also very useful for a quantitative correlation of microglial responses with processes of retinal degeneration [34–36].

## Methods

### Reagents

XBD173 (emapunil) was obtained by custom synthesis from APAC Pharmaceuticals, Ellicott City, MD21042. XBD173 was dissolved in DMSO.

### RNA isolation and reverse transcription

Total RNA was extracted from murine retinas using the NucleoSpin® RNA Mini Kit (Macherey-Nagel, Dueren, Germany). RNA was quantified spectrophotometrically with a NanoDrop 2000 (Thermo Scientific). First-strand cDNA synthesis was carried out with the Revert Aid H Minus First-strand cDNA Synthesis Kit (Fermentas, K1632).

### Quantitative RT-PCR

cDNA (25 ng) were amplified in 10 μl reaction mixture consisting of 5 μl Fast Start Universal Probe Master (Rox) (Roche), 2 μl of primers (10 μM), 0.375 μl purified water, and 0.125 μl of dual-labeled UPL probe (Roche Applied Science, Basel, Switzerland) with an Applied Biosystems 7900 HT Fast Real-Time PCR system (Applied Biosystems, Carlsbad, CA, USA). The following reaction parameters were used: 10 min 95 °C hold, followed by 40 cycles of 15 s 95 °C melt, and 1 min 60 °C anneal/extension. Primer sequences and UPL probe numbers were as follows: *Cd68*, forward primer 5′-ctctctaaggctacaggctgct-3′, reverse primer 5′-tcacggttgcaagagaaaca-3′, probe #27; *Amwap*, forward primer 5′-tttgatcactgtggggatga-3′, reverse primer 5′-acactttctggtgaaggcttg-3′, probe #1; *Tspo*, forward primer 5′-actgtattcagccatggggta-3′, reverse primer 5′-accatagcgtcctctgtgaaa-3′, probe #33; *Il6*, forward primer 5′-gatggatgctaccaaactggat-3′, reverse primer 5′-ccaggtagctatggtactccaga-3′, probe #6; *iNos*, forward primer 5′-ctttgccacggacgagac-3′, reverse primer 5′-tcattgtactctgagggctga-3′, probe #13; *Ccl2*, forward primer 5′-catccacgtgttggctca-3′, reverse primer 5′-gatcatcttgctggtgaatgagt-3′, probe #62; *Casp8*, forward primer 5′-tgaacaatgagatccccaaat-3′, reverse primer 5′-caaaaatttcaagcaggctca-3′, probe #11; and *Atp5b*, forward primer 5′-ggcacaatgcaggaaagg-3′, reverse primer 5′-tcagcaggcacatagatagcc-3′, probe #77. Measurements were performed in triplicates. *Atp5b* expression was used as reference gene, and results were analyzed with the ABI sequence detector software version 2.4 using the ΔΔCt method for relative quantification.

### Animals

Experiments were performed with 10–14-week-old albino BALB/cJ mice and *Cx3cr1*^GFP/+^ mice on C57BL/6J background of both sexes. Animals were housed in an air-conditioned environment with 12-h light-dark schedule and had free access to water and food. All experimental procedures complied with the German law on animal protection and the ARVO Statement for the Use of Animals in Ophthalmic and Vision Research. The animal protocols used in this study were reviewed and approved by the governmental body responsible for animal welfare in the state of Nordrhein-Westfalen (Landesamt für Natur, Umwelt und Verbraucherschutz Nordrhein-Westfalen, Germany) (reference number 84-02.04.2015-A039) and by the Animal Welfare and Ethical Review Board of Queen’s University Belfast under the regulation of the UK Home Office Animal (Scientific Procedures) Act 1986.

### XBD173 administration

The mice received intraperitoneal injections of XBD173 at a dose of 10 mg/kg body weight, dissolved in DMSO or DMSO vehicle control twice daily for the first 2 days, starting 1 day before the light exposure and once daily for the remaining 3 days.

### Light exposure regimens

BALB/cJ and *Cx3cr1*^GFP/+^ mice were dark-adapted for 16 h before light exposure. After pupil dilatation with 1 % phenylephrine and 2.5 % tropicamide under dim red light, the mice were exposed to bright white light with an intensity of 15,000 lux for 1 h or focal white light with an intensity of 50,000 lux delivered by an otoscope (1218AA, Karl Storz, Tuttlingen, Germany) for 10 min, respectively. After light exposure, the animals were housed in dark-reared conditions overnight and then maintained under normal light conditions for the remaining experimental period.

### Immunohistochemistry

Eyes were harvested for immunohistochemical analysis 4 days after light exposure. After fixation with 4 % paraformaldehyde, eyes were embedded in optimal cutting temperature compound or dissected for retinal flat mount analysis. Sixteen-micrometer sections were rehydrated with phosphate-buffered saline (PBS) and blocked with 1 % dried milk solution containing 0.01 % Triton X-100. Flat mounts were incubated with 5 % Tween and 5 % Triton X-100 in PBS overnight, and non-specific binding was blocked by incubation with dried milk solution. Subsequently, retinal sections and flat mounts were incubated with primary antibodies at 4 °C overnight. Primary antibodies targeting the following proteins were used: rabbit anti-Iba1 antibody (dilution 1:500; Wako Chemicals, Neuss, Germany), rabbit anti-TSPO antibody (dilution 1:250; Abcam, Cambridge, UK), rabbit anti-glial fibrillary acidic protein (dilution 1:200; G9269, Sigma, USA), mouse anti-glutamine synthetase (dilution 1:200; MAB302, Millipore, Darmstadt, Germany), and rabbit anti-cone arrestin (Millipore, Darmstadt, Germany). After a washing step, the sections and flat mounts were incubated with a secondary antibody either conjugated to Alexa488 (green; dilution 1:1000) or Alexa594 (red; dilution 1:800) (Jackson Immuno-Research, West Grove, PA, USA) for 1 h. After counterstaining with 4′,6-Diamidin-2-phenylindol (DAPI) in some instances, the samples were mounted in DAKO fluorescent mounting medium (Dako Deutschland GmbH, Hamburg, Germany) and analyzed with an Axioskop2 MOT Plus Apotome microscope (Carl Zeiss) or an Eclipse TE200-U confocal microscope (Nikon).

### Optical coherence tomography

Animals were anesthetized by intraperitoneal injection of Rompun (10 mg/kg body weight)-Ketavet (100 mg/kg body weight), and their pupils were dilated with phenylephrine HCl (0.25 %)-tropicamide (0.05 %) before image acquisition. Spectral domain optical coherence tomography (SD-OCT) was performed on both eyes with a Spectralis™ HRA + OCT device (Heidelberg Engineering) to investigate structural changes in the retina after light exposure and XBD173 administration. Thickness measurements were performed using the Heidelberg Eye Explorer Software using a circular ring scan (circle diameters 3 and 6 mm), centered on the optic nerve head, which represents the average retinal thickness (μm) in a certain field.

### TUNEL assay and morphometric analyses

Retinal sections were labeled with an in situ cell death detection kit, fluorescein (Roche), to detect the amount of apoptotic cells 4 days after light exposure. For a better overview, the sections were also counterstained with DAPI for 10 min. After a washing procedure, sections were mounted in DAKO fluorescent mounting medium (Dako Deutschland GmbH, Hamburg, Germany) and analyzed with an Axioskop2 MOT Plus Apotome microscope (Carl Zeiss). Quantitative morphometric analyses were performed by counting the number of rows of photoreceptor nuclei along the nasal/temporal axis.

### Flow cytometry

Mouse retinas were dissected 4 days after light exposure and dissociated using the Neuronal Tissue Dissociation Kit-Postnatal Neurons (MACS, Miltenyi, Bergisch Gladbach, Germany). To identify microglia cells and macrophages, the single-cell suspension was stained with anti-mouse/human CD11b-APC antibody (MACS, Miltenyi, Bergisch Gladbach, Germany) at a dilution of 1:10 for 15 min in the dark at 4 °C. After a washing step, cells were fixed with FluoroFix Buffer (Biolegend, San Diego, CA, USA) in the dark for 30 min at room temperature. Afterwards, the cells were washed and resuspended in PBS solution (pH 7.2, 0.5 % containing bovine serum albumin (BSA), and 2 mM EDTA) until flow cytometric analyses with a fluorescence-activated cell sorting (FACS) Canto II (Becton Dickinson, Heidelberg, Germany). The number of CD11b+ cells was determined using FlowJo software (Treestar, Ashland, USA).

### Statistical analysis

The differences between control mice and animals after light exposure that either received sham injections or XBD173 injections were analyzed using a one-way ANOVA and Dunnett’s multiple comparison test. *p* < 0.05 was considered statistically significant.

## Results

### The TSPO ligand XBD173 prevents microglia reactivity and gliosis in murine retinas exposed to acute white light

We have previously identified that *Tspo* mRNA and protein are highly induced in genetic models of retinal degeneration and that its specific ligand XBD173 has potent anti-inflammatory activity on microglia in vitro [16]. Based on these data, we hypothesized that XBD173 could also modulate microglial responses in the damaged retina in vivo. We have chosen acute light-induced degeneration as it is a fast and reproducible mouse model that mimics several features of human retinal degenerative diseases including innate immune activation and selective cell death of photoreceptor cells [33].

**Fig. 1:**
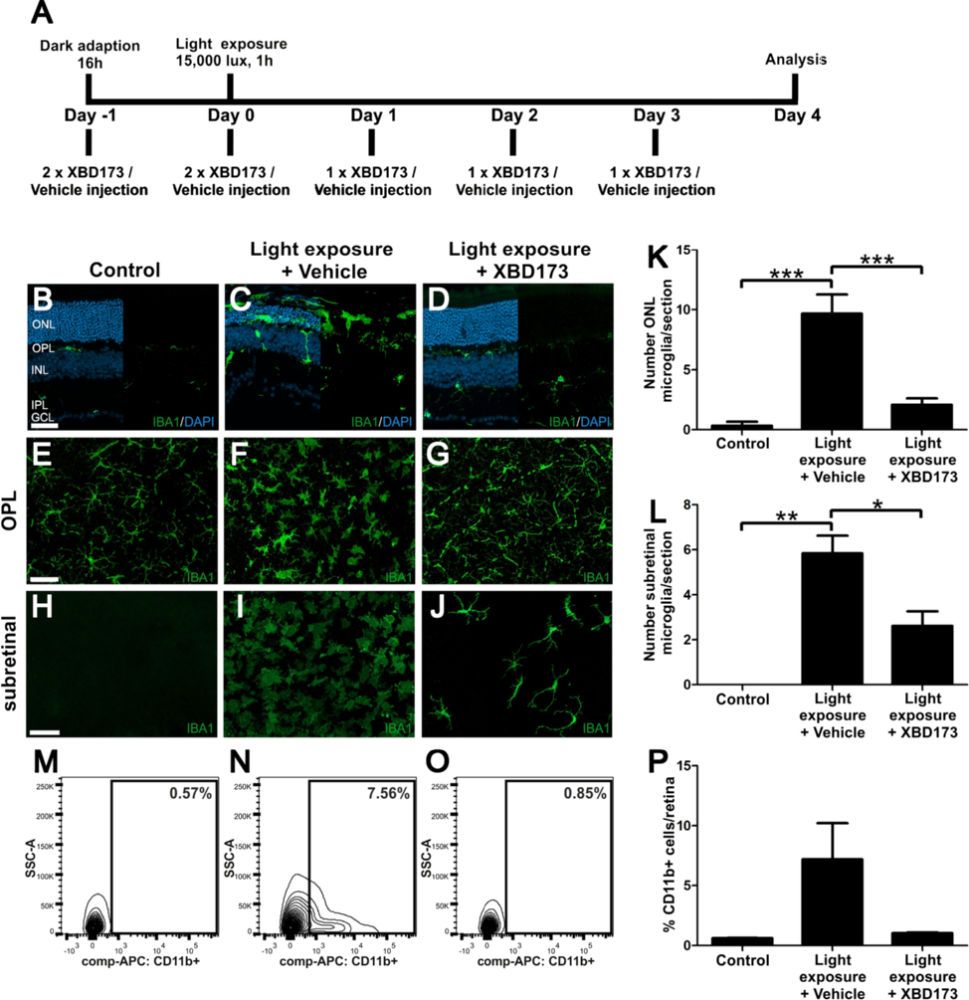
XBD173 treatment of light-exposed mice prevents microglia reactivity. **a** Light exposure regimen and mode of XBD173 administration. Representative photomicrographs show retinal sections (**b**–**d**) and flat mounts (**e**–**j**) stained with IBA1 (*green*) and DAPI (*blue*). In control retinas, microglial cells were located in the OPL, IPL, and GCL (**b**, **e**, **h**). Light-exposed retinas injected with vehicle control showed a massive thinning of the ONL and many amoeboid-shaped, reactive microglia in the ONL and the subretinal space (**c**, **f**, **i**). Compared to vehicle controls, the ONL of XBD173-treated retinas appeared markedly preserved and less amoeboid microglia were detectable in the ONL and the subretinal space (**d**, **g**, **j**). The total number of Iba1-positive microglial cells in the ONL (**k**) and the subretinal area (**l**) after light damage was significantly reduced in the XBD173 therapy group. Data show mean ± SEM (control *n* = 3, light exposure and vehicle treatment *n* = 6, light exposure and XBD173 treatment *n* = 12 sections). The percentage of CD11b-positive cells in the retina as determined by flow cytometry was strongly reduced by XBD173 treatment in representative FACS plots (**m**–**o**) and quantitative analyses of *n* = 3–4 retinas (**p**). *ONL* outer nuclear layer, *OPL* outer plexiform layer, *INL* inner nuclear layer, *IPL* inner plexiform layer, *GCL* ganglion cell layer. *p < 0.05; **p < 0.01; ***p < 0.001. Scale bar 50 μm

Dark-adapted BALB/cJ mice were exposed to white light with an intensity of 15,000 lux for 1 h. The animals received daily intraperitoneal injections of 10 mg/kg XBD173 or vehicle, starting 1 day before the light exposure for five consecutive days (Fig. 1a). Four days after light exposure, the effects of XBD173 treatment on microgliosis were analyzed by staining of retinal sections and flat mounts. In healthy controls, immunolabeling of retinal sections with the marker IBA1 showed ramified microglia exclusively in the plexiform layers and inner retina (Fig. 1b). Light exposure and vehicle administration induced strong microglia reactivity resulting in the migration and appearance of amoeboid-shaped microglia in the degenerating photoreceptor layer and the subretinal area (Fig. 1c). This severe thinning of the outer retina and accumulation of microglia in and around the outer nuclear layer was not detectable in mice treated with XBD173 (Fig. 1d). We next analyzed changes in the microglial network in retinal flat mounts stained with Iba1. In contrast to controls, where microglial cells are highly ramified in the outer plexiform layer (OPL) (Fig. 1e), light-exposed retinas showed clear signs of microglia reactivity in the OPL (Fig. 1f), which was reverted in conditions of XBD173 therapy (Fig. 1g). Furthermore, the appearance of many amoeboid-shaped microglia in the subretinal area triggered by light exposure was strongly reduced in the XBD173 treatment group (Fig. 1h–j). This finding that XBD173 very likely prevented migration of many microglia from the inner to the outer retina was corroborated by a quantitative analysis counting the number of microglial cells in the outer nuclear layer (ONL) and subretinal area (Fig. 1k, l). We then determined the number of CD11b-positive cells in the retina using flow cytometry. These data also showed that XBD173 administration strongly reduced the percentage of CD11b-positive cells in the light-exposed retina compared to vehicle controls (Fig. 1m–p).

**Fig. 2:**
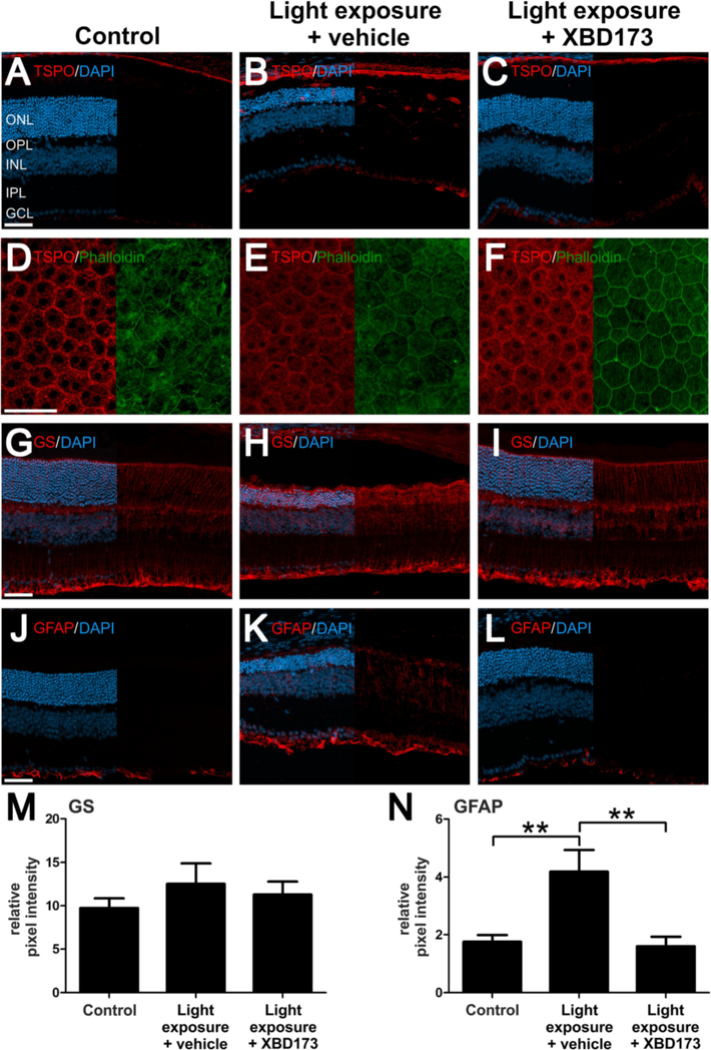
XBD173 treatment of light-exposed mice down-regulates TSPO in microglia and prevents gliosis. Representative photomicrographs of retinal sections and flat mounts from mice 4 days after light exposure. Control retinas show weak TSPO labeling (**a**), and strong up-regulation of TSPO expression confined to microglia was present upon light exposure and vehicle treatment (**b**). XBD173-treated mice displayed only constitutive expression of TSPO in the RPE, but no strong signal in the retina was found (**c**). Number of analyzed photomicrographs: control *n* = 5 sections from two individual mice, light exposure and vehicle treatment *n* = 7 sections from five individual mice, light exposure and XBD173 treatment *n* = 11 sections from five individual mice. The expression of TSPO in the RPE was relatively unaffected in the different conditions as shown in flat mount images stained with anti-TSPO antibody and phalloidin (**d**–**f**). Glutamine synthetase (*GS*), a constitutive Müller cell protein, and glial fibrillary acidic protein (*GFAP*) as markers for gliosis were analyzed (**g**–**n**). GS expression did not differ in the various treatment groups (**g**–**i**, **m**) whereas GFAP expression was changed significantly (**j**–**l**, **n**). Data show mean ± SEM (control *n* = 5 sections from five individual mice, light exposure and vehicle treatment *n* = 5 sections from three individual mice, light exposure and XBD173 treatment *n* = 5 sections from four individual mice). **p < 0.01. *ONL* outer nuclear layer, *OPL* outer plexiform layer, *INL* inner nuclear layer, *IPL* inner plexiform layer, *GCL* ganglion cell layer. Scale bar 50 μm

We then wanted to better characterize the reactive gliosis and its relation to the expression of TSPO itself. Exposure to light in the vehicle control triggered strong expression of TSPO that was mainly confined to outer retinal microglia and some astrocytes in the ganglion cell layer (Fig. 2a, b). This cellular expression pattern is in agreement with previous immunohistochemical data from genetic mouse models of retinal degeneration [16, 37] and highlights the induced expression of TSPO in microglia upon activation. Administration of XBD173 strongly diminished TSPO expression (Fig. 2c), indicating a much less reactive microgliosis and confirming our stainings with Iba1 (Fig. 1). Interestingly, we also identified specific expression of TSPO in the retinal pigment epithelium (RPE) network that was visualized by co-staining with phalloidin (Fig. 2d). However, this RPE-specific expression pattern of TSPO was not obviously influenced by light exposure or XBD173 therapy (Fig. 2e, f), indicating a constitutive role of TSPO in the RPE that is unrelated to gliosis. We then analyzed the expression of glutamine synthetase (GS), a constitutively expressed Müller cell protein and glial fibrillary acid protein (GFAP), a marker für Müller cell and astrocyte reactivity. GS staining was not significantly changed by light exposure and XBD173 treatment (Fig. 2g–i, m), whereas XBD173 significantly reduced the light-dependent induction of GFAP expression (Fig. 2j–l, n). These findings indicate that gliosis triggered by light damage can be prevented by targeting TSPO.

### XBD173 reduces pro-inflammatory gene expression in retinal degeneration

**Fig. 3:**
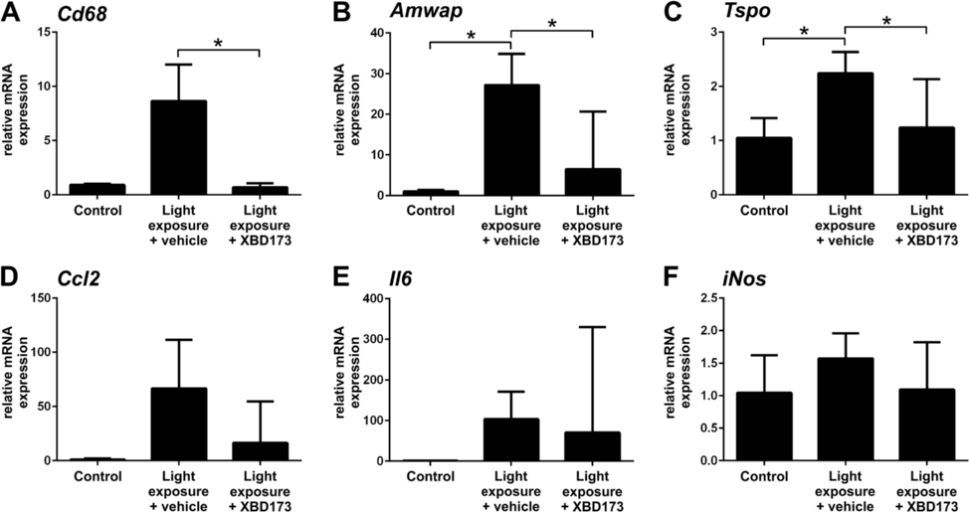
XBD173 treatment dampens microglia-related pro-inflammatory marker expression after light exposure. To determine the mRNA expression of inflammation-associated genes in the retina, qRT-PCR analysis was performed 4 days after light exposure. **a**–**c** The microglia markers *Cd68* and *Amwap* were up-regulated after light exposure, and TSPO levels were also increased. In contrast, the retinas from XBD173-treated mice expressed significantly less *Cd68*, *Amwap*, and *Tspo* transcripts after light exposure. **d**, **e** XBD173 treatment reduced the expression of *Ccl2* and *Il6* that were elevated by light exposure. **f** iNOS expression was not altered. Data show mean ± SEM out of three independent experiments (control *n* = 4–8, light exposure plus vehicle treatment *n* = 8–15, light exposure plus XBD173 treatment *n* = 8–15 per group, measured in duplicates) with *p < 0.05; **p < 0.01; ***p < 0.001

In addition to these cellular analyses of retinal microglia, we investigated whether mRNA levels of pro-inflammatory transcripts were influenced by XBD173 administration in vivo. CD68, activated microglia/macrophage whey acidic protein (AMWAP), and TSPO itself are molecules that are connected with microglia proliferation and reactivity [16, 38, 39]. Four days after light exposure, all three genes were induced in the retina, with especially high levels of *Amwap* (Fig. 3a–c). In light-exposed mice treated with XBD173, the expression of *Cd68* (Fig. 3a, *p* = 0.0342), *Amwap* (Fig. 3b, *p* = 0.0182), and *Tspo* (Fig. 3c, *p* = 0.0338) were significantly suppressed. Furthermore, transcripts that reflect the activation of key microglial pathways including chemotaxis, pro-inflammatory cytokines, and radical production were analyzed. mRNA levels of the chemotactic molecule *Ccl2* and the cytokine *Il6* were strongly up-regulated in the retinas of sham-injected light-exposed mice, and XBD173 administration diminished their expression (Fig. 3d, e). Notably, the expression of *iNos* was not significantly altered by light exposure or XBD173 treatment (Fig. 3f) potentially indicating different time kinetics of chemokine/cytokine levels and oxygen radical responses. These data suggest that XBD173 has potent microglia-related immuno-modulatory effects in vivo in the mouse retina.

### XBD173 treatment prevents light-induced retinal degeneration

**Fig. 4:**
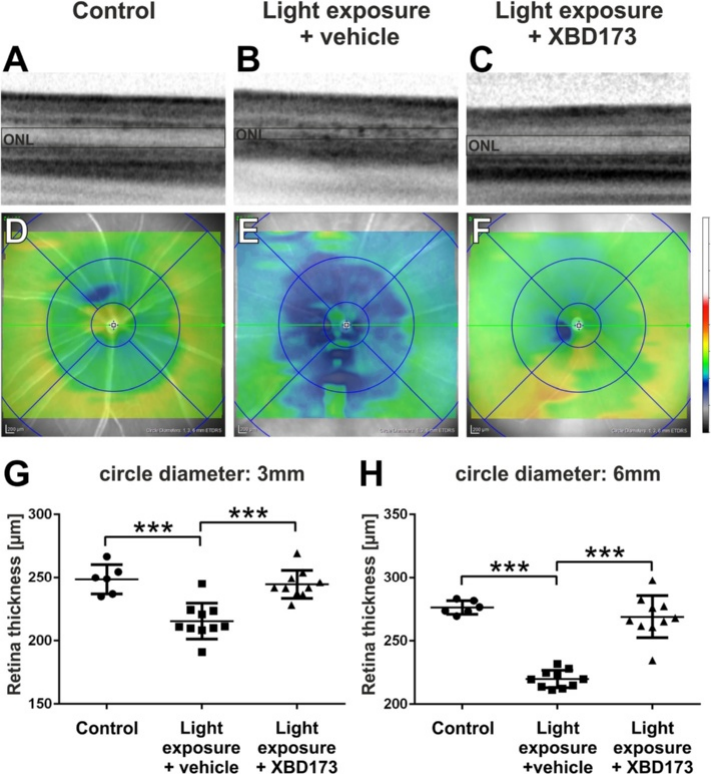
XBD173 preserves retinal thickness in light-exposed mice. SD-OCT was performed 4 days after light exposure to analyze changes in retinal structures. **a**–**c** Light-exposed mice show an altered reflectance in the ONL, which was not present in XBD173-treated mice. **d**–**f** Representative heat maps show the average retinal thickness of control and light-exposed mice after vehicle or XBD173 treatment, respectively. Light-exposed mice show a significant thinning of the retina, in the central (**g**) and more peripheral area (**h**), which was preserved by XBD173 treatment. **g**, **h** One data point represents the average thickness of the central retina, calculated from four different areas around the optic nerve head in circle diameters of 3 mm (**g**) and 6 mm (**h**), respectively. Data show mean ± SEM out of two independent experiments (control *n* = 6, light exposure plus vehicle treatment *n* = 10, light exposure plus XBD173 treatment *n* = 10/group) with ***p < 0.001

We next asked whether targeting TSPO with XBD173 also improves the outcome of disease progression. We first performed in vivo optical coherence tomography (OCT) to detect the retinal damage after light exposure and under conditions of XBD173 treatment. The OCT images showed clear changes especially in ONL reflectance in the retinas of sham-treated light-exposed mice, indicating a severe degeneration of the photoreceptor layer (Fig. 4a, b). In contrast, XBD173-treated animals displayed nearly normal retinal layers similar to that of healthy controls (Fig. 4c). Volume scans revealed a significant thinning of the retinal tissue, especially in the central area around the optic nerve head after light exposure, which was much less pronounced in the XBD173-treated groups (Fig. 4d–f). Quantification of the retinal thickness in all analyzed animals demonstrates a significant reduction in the 3- and 6-mm central areas, respectively, after light exposure (*p* < 0.0001), that could be rescued by administration of XBD173 (*p* < 0.0001) (Fig. 4g, h).

**Fig. 5:**
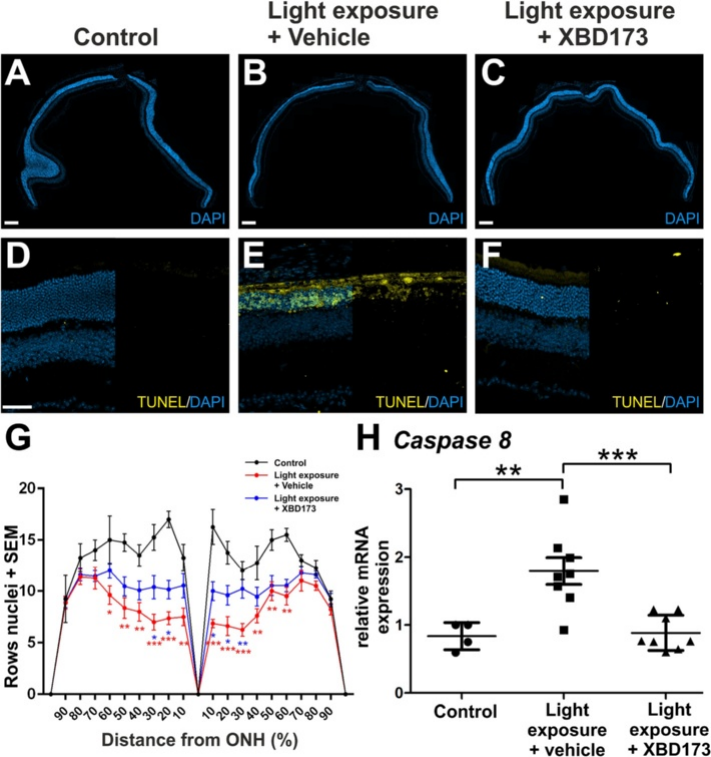
XBD173 protects the light-exposed retina from cell death. **a**–**c** Representative panorama sections of the retina were stained with DAPI to further characterize structural changes. Light exposure caused severe degeneration particularly of the ONL. XBD173 administration reduced degeneration, represented by a clearly thicker photoreceptor layer, scale bar 200 μM. **d**–**f** Representative photomicrographs of TUNEL-stained retinal sections show the amount of apoptotic cells 4 days after light exposure. Light exposure caused a strong increase of apoptotic cells, especially in the ONL. Only very few TUNEL-positive cells were visible after XBD173 administration. Scale bar 50 μm (**g**). For quantification of retinal thickness, anterior and posterior areas were divided into ten sections with the optic nerve as reference and rows of photoreceptor nuclei were counted. Data show mean ± SEM (control *n* = 4, light exposure plus vehicle treatment *n* = 8, light exposure plus XBD173 treatment *n* = 10/group) with *p < 0.05; **p < 0.01; ***p < 0.001 light exposed + vehicle versus control (*red color*) and light exposed + XBD173 versus light exposed + vehicle (*blue color*). **h** mRNA analyses revealed a significantly lower expression of caspase 8 in light-exposed XBD173-treated mice compared to controls and vehicle-injected retinas. Data show mean ± SEM out of two independent experiments (control *n* = 4, light exposure plus vehicle treatment *n* = 8, light exposure plus XBD173 treatment *n* = 8/group, measured in triplicates) with **p < 0.01; ***p < 0.001

To confirm these findings on the histological level, panorama images of retinal sections proceeding through the optic nerve head obtained 4 days after light exposure were stained with DAPI (Fig. 5a–c). The photoreceptor layer showed a clear thinning in sham-injected mice exposed to light, which was not evident in mice after XBD173 administration (Fig. 5a–c). In accordance with these data, the number of terminal deoxynucleotidyl transferase dUTP nick end labeling (TUNEL)-positive cells as indicators of cell death was also increased with light and strongly reduced by XBD173 injection (Fig. 5d, e). Further quantitative morphometric analyses along the nasal/temporal axis revealed that light exposure especially reduced the central retinal thickness and that this decline was significantly less in the XBD173-treated group of mice (Fig. 5g). Moreover, molecular analyses using quantitative real-time PCR (qRT-PCR) of total retinal RNA detected a clear counter-regulatory effect of XBD173 on caspase 8 mRNA levels (Fig. 5h, *p* = 0.0007). These data together clearly point towards a strong neuroprotective effect of XBD173 in conditions of light damage.

### XBD173 prevents focal light-induced retinal damage

**Fig. 6:**
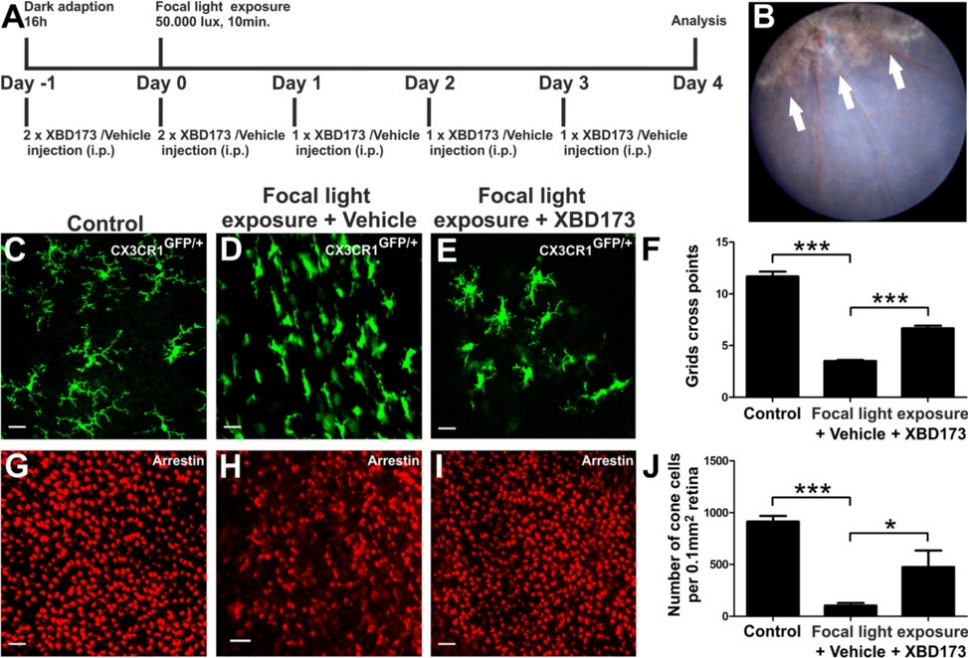
XBD173 treatment prevents microglia reactivity and rescues cone photoreceptors in focal light-induced damage. **a** Light exposure regimen and mode of XBD173 administration. **b** Representative fundus image showing the focal light lesion 4 days after *Cx3cr1* ^GFP/+^ mice were exposed to 50,000 lux for 10 min. **c**–**e** Representative confocal images of GFP-positive retinal microglia in flat mounts from control mice (**c**) and lesion areas of light-exposed mice (**d**, **e**). The average size of microglial cells was assessed using the grid cross point system (**f**). Data show mean ± SEM (*n* = 40–180 cells per group) with ***p < 0.001. **g**–**i** Retinal flat mounts were processed for cone arrestin staining and examined by confocal microscopy. Representative confocal images of cone arrestin-positive cells in flat mounts from control mice (**g**) and lesion areas of light-exposed mice (**h**, **i**). **j** Average density of cone photoreceptor cells. Data show mean ± SEM (*n* = 6–8 retinas per group) with *p < 0.05; ***p < 0.001. Scale bar 50 μm

We were finally interested to validate our findings in an independent model using focal light-induced retinal damage. *Cx3cr1*^GFP/+^ reporter mouse eyes, which facilitate label-free microglia visualization, were exposed to 50,000 lux cold light delivered by an otoscope for 10 min in the same experimental design as described above for complete light exposure (Fig. 6a). Fundus imaging showed a clear focal light-induced retinal damage with a central area of atrophy (Fig. 6b). Confocal imaging of GFP-positive ONL microglia within the retinal lesion area of sham-treated mice clearly indicated a reactive phenotype compared to controls and XBD173-injected animals, respectively (Fig. 6c–e). As short cellular processes and enlargement of soma are characteristic for microglial activation, we quantified this morphological change using a grid system by counting the points of all cells that crossed the grid as described previously [40]. This analysis showed a significant reduction of grid cross points in focal light-exposed retinas, and this was fully reverted in the XBD173-treated group of animals (Fig. 6f).

In the last set of experiments, we determined the number of cone photoreceptors in the focal light damage model using confocal microscopy of retinal flat mounts that were stained with anti-cone arrestin antibodies. The areas of focal light damage showed a much weaker arrestin staining compared to controls and compared to the retinas of XBD173-treated animals, respectively (Fig. 6g–i). The quantitative analysis of 6–8 independent retinas revealed a more than 8-fold reduction in the number of cone photoreceptors upon light damage and a 4.8-fold increase in the XBD173 therapy group, indicating a prominent effect on photoreceptor survival (Fig. 6j).

## Discussion

Due to its high expression in activated glia, TSPO is a marker for brain gliosis and TSPO ligands are commonly used for in vivo imaging in humans and mice [41, 42]. We have previously identified high TSPO levels in retinal microglia of murine models of inherited retinal degeneration [16, 37]. In the present study, we now show that TSPO is also strongly induced in retinal microglia after light exposure and that the TSPO ligand XBD173 has potent microglia-modulatory and neuroprotective functions in the retina in vivo. To our knowledge, this is the first report of TSPO-directed pharmacological targeting of the diseased retina.

To mimic exposure to bright daylight, which is a well-known environmental risk factor for retinal degenerations, we exposed the mice to 15,000 lux UV-free white light for 1 h in the first set of experiments and to 50,000 lux focal cold white light for 10 min in the validation studies. White light has an emission spectrum relatively similar to that of daylight, and it is less artificial than the light of a particular wavelength [43, 44]. Furthermore, white light contains a significant fraction of short-wavelength blue light (403 nm) which is thought to have a higher damaging potential than the light of longer wavelength including green light (490–580 nm), which was used in some studies to mimic retinal degeneration [45]. The higher damaging potential of blue light is due to a process called photo-reversal, the regeneration of rhodopsin from bleaching intermediates that results in a higher number of photon absorption in a certain time span [46].

Very early microglial activation was observed already 1 day after light challenge in both models (data not shown), indicating that microglia sense subtle disturbances in the light-damaged retina before overt cell death occurs. TSPO expression was present in these reactive microglia, and our analyses towards the phenotype and behavior of microglia together with previous in vitro data [16] suggest that XBD173 exerts its neuroprotective function at least partially via modulation of microglia. Likewise, another specific TSPO ligand, etifoxine, also potently diminished inflammatory pathology and thereby attenuated the clinical score of experimental autoimmune encephalomyelitis, an experimental mouse model for multiple sclerosis [25]. The tight correlation of microglia reactivity with TSPO expression was also identified in the genetic mouse model of myosin VII deficiency that mimics both features of retinal dystrophies and glaucoma [47]. Therefore, TSPO induction in retinal microglia may be an early and reliable marker for changes in the microglia phenotype in different types of retinal degenerative diseases [37].

Our mRNA expression data of XBD173-treated retinas showed not only decreased transcript levels of *Cd68*, *Amwap*, and *Tspo* but also reduced *Ccl2* and *Il6* expression. The CCL2/CCR2 axis is crucial in subretinal macrophage and microglia accumulation in retinal degeneration models and human AMD, and these findings implicate that CCL2/CCR2 inhibition may be a novel tool to limit inflammation and neurodegeneration in the retina [48, 49]. Similarly, human reactive microglia express IL6, which in turn prevents retinal regeneration and promotes subretinal immune cell survival [50, 51]. Thus, the XBD173/TSPO axis seems to target two key pathways of chronic microglia reactivity in the retina.

As we have shown previously in microglial cell cultures [16], one potential mechanism of the anti-inflammatory effects of XBD173 could be the local synthesis of pregnenolone, as has been also demonstrated for brain astrocytes [52]. Pregnenolone can be further metabolized to progesterone and allopregnanolone that are both potent neuroprotective and anti-inflammatory molecules [53–55]. When orally applied to *rd1* mice with inherited retinal degeneration, progesterone potently reduced oxidative stress levels, diminished gliosis, and provided a temporal improvement in photoreceptor function [56].

We have demonstrated constitutive mitochondrial TSPO expression in the RPE that was unaffected by light exposure or XBD173 therapy. Therefore, TSPO in RPE mitochondria could potentially fulfill a housekeeping function. Of note, increased damage of mitochondrial DNA specifically in the RPE has been recently implicated as a risk factor for AMD and mutations of electron transport chain components could potentially limit energy production [57]. Thus, in addition to its anti-inflammatory effects on microglia, TSPO may sustain mitochondrial homeostasis and integrity by regulating the oxygen consumption rate. However, this function and the previously identified physiological roles of TSPO remain to be characterized with novel cell-type specific knockout mouse models [58, 59].

## Conclusions

We have shown that modulation of microglia with the synthetic TSPO ligand XBD173 preserved the retinal structure by counter-regulation of microglial pro-inflammatory responses during light exposure. Our data suggest that targeting TSPO in the retina may be a novel promising approach for anti-inflammatory and neuroprotective therapies in retinal degenerative disorders.

## References

[1] Hume DA, Perry VH, Gordon S (1983). Immunohistochemical localization of a macrophage-specific antigen in developing mouse retina: phagocytosis of dying neurons and differentiation of microglial cells to form a regular array in the plexiform layers. J Cell Biol.

[2] Kettenmann H, Hanisch UK, Noda M, Verkhratsky A (2011). Physiology of microglia. Physiol Rev.

[3] Damani MR, Zhao L, Fontainhas AM, Amaral J, Fariss RN, Wong WT (2011). Age-related alterations in the dynamic behavior of microglia. Aging Cell.

[4] Nimmerjahn A, Kirchhoff F, Helmchen F (2005). Resting microglial cells are highly dynamic surveillants of brain parenchyma in vivo. Science.

[5] Aloisi F (2001). Immune function of microglia. Glia.

[6] Gupta N, Brown KE, Milam AH (2003). Activated microglia in human retinitis pigmentosa, late-onset retinal degeneration, and age-related macular degeneration. Exp Eye Res.

[7] Langmann T (2007). Microglia activation in retinal degeneration. J Leukoc Biol.

[8] Karlstetter M, Langmann T (2014). Microglia in the aging retina. Adv Exp Med Biol.

[9] Xu H, Chen M, Forrester JV (2009). Para-inflammation in the aging retina. Prog Retin Eye Res.

[10] Zhao L, Zabel MK, Wang X, Ma W, Shah P, Fariss RN, Qian H, Parkhurst CN, Gan WB, Wong WT (2015). Microglial phagocytosis of living photoreceptors contributes to inherited retinal degeneration. EMBO Mol Med.

[11] Sierra A, Gottfried-Blackmore AC, McEwen BS, Bulloch K (2007). Microglia derived from aging mice exhibit an altered inflammatory profile. Glia.

[12] Roque RS, Rosales AA, Jingjing L, Agarwal N, Al-Ubaidi MR (1999). Retina-derived microglial cells induce photoreceptor cell death in vitro. Brain Res.

[13] Wu DC, Jackson-Lewis V, Vila M, Tieu K, Teismann P, Vadseth C, Choi DK, Ischiropoulos H, Przedborski S (2002). Blockade of microglial activation is neuroprotective in the 1-methyl-4-phenyl-1,2,3,6-tetrahydropyridine mouse model of Parkinson disease. J Neurosci.

[14] Amor S, Puentes F, Baker D, van der Valk P (2010). Inflammation in neurodegenerative diseases. Immunology.

[15] Chen M, Xu H. Parainflammation, chronic inflammation, and age-related macular degeneration. J Leukoc Biol. 2015. 10.1189/jlb.3RI0615-239R PMC4733662 26292978

[16] Karlstetter M, Nothdurfter C, Aslanidis A, Moeller K, Horn F, Scholz R, Neumann H, Rupprecht R, Langmann T (2014). Translocator protein (18 kDa) (TSPO) is expressed in reactive retinal microglia and modulates microglial inflammation and phagocytosis. J Neuroinflammation.

[17] Wang M, Wang X, Zhao L, Ma W, Rodriguez IR, Fariss RN, Wong WT (2014). Macroglia-microglia interactions via TSPO signaling regulates microglial activation in the mouse retina. J Neurosci.

[18] Papadopoulos V, Liu J, Culty M (2007). Is there a mitochondrial signaling complex facilitating cholesterol import?. Mol Cell Endocrinol.

[19] Kuhlmann AC, Guilarte TR (2000). Cellular and subcellular localization of peripheral benzodiazepine receptors after trimethyltin neurotoxicity. J Neurochem.

[20] Maeda J, Higuchi M, Inaji M, Ji B, Haneda E, Okauchi T, Zhang MR, Suzuki K, Suhara T (2007). Phase-dependent roles of reactive microglia and astrocytes in nervous system injury as delineated by imaging of peripheral benzodiazepine receptor. Brain Res.

[21] Veiga S, Azcoitia I, Garcia-Segura LM (2005). Extragonadal synthesis of estradiol is protective against kainic acid excitotoxic damage to the hippocampus. Neuroreport.

[22] Girard C, Liu S, Adams D, Lacroix C, Sineus M, Boucher C, Papadopoulos V, Rupprecht R, Schumacher M, Groyer G (2012). Axonal regeneration and neuroinflammation: roles for the translocator protein 18 kDa. J Neuroendocrinol.

[23] Chen MK, Guilarte TR (2008). Translocator protein 18 kDa (TSPO): molecular sensor of brain injury and repair. Pharmacol Ther.

[24] Barron AM, Garcia-Segura LM, Caruso D, Jayaraman A, Lee JW, Melcangi RC, Pike CJ (2013). Ligand for translocator protein reverses pathology in a mouse model of Alzheimer’s disease. J Neurosci.

[25] Daugherty DJ, Selvaraj V, Chechneva OV, Liu XB, Pleasure DE, Deng W (2013). A TSPO ligand is protective in a mouse model of multiple sclerosis. EMBO Mol Med.

[26] Wei XH, Wei X, Chen FY, Zang Y, Xin WJ, Pang RP, Chen Y, Wang J, Li YY, Shen KF (2013). The upregulation of translocator protein (18 kDa) promotes recovery from neuropathic pain in rats. J Neurosci.

[27] Rupprecht R, Rammes G, Eser D, Baghai TC, Schule C, Nothdurfter C, Troxler T, Gentsch C, Kalkman HO, Chaperon F (2009). Translocator protein (18 kD) as target for anxiolytics without benzodiazepine-like side effects. Science.

[28] Nothdurfter C, Rammes G, Baghai TC, Schule C, Schumacher M, Papadopoulos V, Rupprecht R (2012). Translocator protein (18 kDa) as a target for novel anxiolytics with a favourable side-effect profile. J Neuroendocrinol.

[29] Nothdurfter C, Baghai TC, Schule C, Rupprecht R (2012). Translocator protein (18 kDa) (TSPO) as a therapeutic target for anxiety and neurologic disorders. Eur Arch Psychiatry Clin Neurosci.

[30] Rupprecht R, Papadopoulos V, Rammes G, Baghai TC, Fan J, Akula N, Groyer G, Adams D, Schumacher M (2010). Translocator protein (18 kDa) (TSPO) as a therapeutic target for neurological and psychiatric disorders. Nat Rev Drug Discov.

[31] Cruickshanks KJ, Klein R, Klein BE (1993). Sunlight and age-related macular degeneration. The beaver dam eye study. Arch Ophthalmol.

[32] Swaroop A, Chew EY, Rickman CB, Abecasis GR (2009). Unraveling a multifactorial late-onset disease: from genetic susceptibility to disease mechanisms for age-related macular degeneration. Annu Rev Genomics Hum Genet.

[33] Grimm C, Reme CE (2013). Light damage as a model of retinal degeneration. Methods Mol Biol.

[34] Marc RE, Jones BW, Watt CB, Vazquez-Chona F, Vaughan DK, Organisciak DT (2008). Extreme retinal remodeling triggered by light damage: implications for age related macular degeneration. Mol Vis.

[35] Narimatsu T, Ozawa Y, Miyake S, Kubota S, Hirasawa M, Nagai N, Shimmura S, Tsubota K (2013). Disruption of cell-cell junctions and induction of pathological cytokines in the retinal pigment epithelium of light-exposed mice. Invest Ophthalmol Vis Sci.

[36] Pennesi ME, Neuringer M, Courtney RJ (2012). Animal models of age related macular degeneration. Mol Aspects Med.

[37] Karlstetter M, Scholz R, Rutar M, Wong WT, Provis JM, Langmann T (2015). Retinal microglia: just bystander or target for therapy?. Prog Retin Eye Res.

[38] Karlstetter M, Walczak Y, Weigelt K, Ebert S, Van den Brulle J, Schwer H, Fuchshofer R, Langmann T (2010). The novel activated microglia/macrophage WAP domain protein, AMWAP, acts as a counter-regulator of proinflammatory response. J Immunol.

[39] Aslanidis A, Karlstetter M, Scholz R, Fauser S, Neumann H, Fried C, Pietsch M, Langmann T (2015). Activated microglia/macrophage whey acidic protein (AMWAP) inhibits NFkappaB signaling and induces a neuroprotective phenotype in microglia. J Neuroinflammation.

[40] Chen M, Zhao J, Luo C, Pandi SP, Penalva RG, Fitzgerald DC, Xu H (2012). Para-inflammation-mediated retinal recruitment of bone marrow-derived myeloid cells following whole-body irradiation is CCL2 dependent. Glia.

[41] Chauveau F, Boutin H, Van Camp N, Dolle F, Tavitian B (2008). Nuclear imaging of neuroinflammation: a comprehensive review of [11C]PK11195 challengers. Eur J Nucl Med Mol Imaging.

[42] Venneti S, Lopresti BJ, Wiley CA (2013). Molecular imaging of microglia/macrophages in the brain. Glia.

[43] Wenzel A, Grimm C, Samardzija M, Reme CE (2005). Molecular mechanisms of light-induced photoreceptor apoptosis and neuroprotection for retinal degeneration. Prog Retin Eye Res.

[44] Youssef PN, Sheibani N, Albert DM (2011). Retinal light toxicity. Eye (Lond).

[45] Zhang C, Lei B, Lam TT, Yang F, Sinha D, Tso MO (2004). Neuroprotection of photoreceptors by minocycline in light-induced retinal degeneration. Invest Ophthalmol Vis Sci.

[46] Grimm C, Wenzel A, Williams T, Rol P, Hafezi F, Reme C (2001). Rhodopsin-mediated blue-light damage to the rat retina: effect of photoreversal of bleaching. Invest Ophthalmol Vis Sci.

[47] Schubert T, Gleiser C, Heiduschka P, Franz C, Nagel-Wolfrum K, Sahaboglu A, Weisschuh N, Eske G, Rohbock K, Rieger N (2015). Deletion of myosin VI causes slow retinal optic neuropathy and age-related macular degeneration (AMD)-relevant retinal phenotype. Cell Mol Life Sci.

[48] Raoul W, Auvynet C, Camelo S, Guillonneau X, Feumi C, Combadiere C, Sennlaub F (2010). CCL2/CCR2 and CX3CL1/CX3CR1 chemokine axes and their possible involvement in age-related macular degeneration. J Neuroinflammation.

[49] Sennlaub F, Auvynet C, Calippe B, Lavalette S, Poupel L, Hu SJ, Dominguez E, Camelo S, Levy O, Guyon E (2013). CCR2(+) monocytes infiltrate atrophic lesions in age-related macular disease and mediate photoreceptor degeneration in experimental subretinal inflammation in Cx3cr1 deficient mice. EMBO Mol Med.

[50] Balasubramaniam B, Carter DA, Mayer EJ, Dick AD (2009). Microglia derived IL-6 suppresses neurosphere generation from adult human retinal cell suspensions. Exp Eye Res.

[51] Levy O, Calippe B, Lavalette S, Hu SJ, Raoul W, Dominguez E, Housset M, Paques M, Sahel JA, Bemelmans AP (2015). Apolipoprotein E promotes subretinal mononuclear phagocyte survival and chronic inflammation in age-related macular degeneration. EMBO Mol Med.

[52] Cascio C, Brown RC, Liu Y, Han Z, Hales DB, Papadopoulos V (2000). Pathways of dehydroepiandrosterone formation in rat brain glia. J Steroid Biochem Mol Biol.

[53] Yao XL, Liu J, Lee E, Ling GS, McCabe JT (2005). Progesterone differentially regulates pro- and anti-apoptotic gene expression in cerebral cortex following traumatic brain injury in rats. J Neurotrauma.

[54] Djebaili M, Guo Q, Pettus EH, Hoffman SW, Stein DG (2005). The neurosteroids progesterone and allopregnanolone reduce cell death, gliosis, and functional deficits after traumatic brain injury in rats. J Neurotrauma.

[55] Djebaili M, Hoffman SW, Stein DG (2004). Allopregnanolone and progesterone decrease cell death and cognitive deficits after a contusion of the rat pre-frontal cortex. Neuroscience.

[56] Sanchez-Vallejo V, Benlloch-Navarro S, Lopez-Pedrajas R, Romero FJ, Miranda M (2015). Neuroprotective actions of progesterone in an in vivo model of retinitis pigmentosa. Pharmacol Res.

[57] Terluk MR, Kapphahn RJ, Soukup LM, Gong H, Gallardo C, Montezuma SR, Ferrington DA (2015). Investigating mitochondria as a target for treating age-related macular degeneration. J Neurosci.

[58] Selvaraj V, Stocco DM (2015). The changing landscape in translocator protein (TSPO) function. Trends Endocrinol Metab.

[59] Gut P, Zweckstetter M, Banati RB (2015). Lost in translocation: the functions of the 18-kD translocator protein. Trends Endocrinol Metab.

